# The Prognostic Characteristics and Recurrence Patterns of High Grade Endometrioid Endometrial Cancer: A Large Retrospective Analysis of a Tertiary Center

**DOI:** 10.3390/jcm12093141

**Published:** 2023-04-26

**Authors:** Andreas Zouridis, Kianoush Zarrindej, Joshua Rencher, Christina Pappa, Ammara Kashif, Sarah Louise Smyth, Negin Sadeghi, Alisha Sattar, Stephen Damato, Federico Ferrari, Antonio Simone Laganà, Mostafa Abdalla, Sean Kehoe, Susan Addley, Hooman Soleymani majd

**Affiliations:** 1 Oxford University Hospitals NHS Foundation Trust, Oxford OX3 7LE, UK; andreas.e.zouridis@gmail.com (A.Z.);; 2 Buckinghamshire NHS Foundation Trust, Bucks HP11 2TT, UK; 3 Royal Berkshire NHS Foundation Trust, Reading RG1 5AN, UK; 4 Department of Clinical and Experimental Sciences, University of Brescia, 25136 Brescia, Italy; federico.ferrari@unibs.it; 5 Unit of Gynecologic Oncology, ARNAS “Civico-Di Cristina-Benfratelli”, Department of Health Promotion, Mother and Child Care, Internal Medicine and Medical Specialties (PROMISE), University of Palermo, 90127 Palermo, Italy; 6 Gynaecology—Guy’s and St Thomas’ NHS Foundation Trust, London SE1 9RT, UK; 7 University Hospitals of Derby and Burton NHS Foundation Trust, Derby DE22 3NE, UK

**Keywords:** endometrioid endometrial cancer, grade 3, high grade, prognosis, recurrence

## Abstract

High grade endometrioid endometrial cancer (HGEEC) is a heterogeneous group of tumors with unclear prognostic features. The aim of the present study is to evaluate the independent risk factors for recurrence and mortality and to describe the recurrence patterns of HGEEC. Ninety-six consecutive cases of HGEEC treated with primary surgery in a single Tertiary Center were retrospectively reviewed. Clinicopathological and treatment details were recorded, and all patients were closely followed up. Disease-free, overall and cancer-specific survival rates were 83.8%, 77.8% and 83.6%, respectively. Cervical stromal involvement was independently related to recurrence (HR = 25.67; 95%CI 2.95–223.30; *p* = 0.003) and cancer-related death (HR = 15.39; 95%CI 1.29–183.43; *p* = 0.031) after adjusting for other pathological and treatment variables. Recurrence rate was 16%, with 60% of these cases having lung metastases and only one case with single vaginal vault recurrence. 81.81% of the recurrences presented with symptoms and not a single recurrence was diagnosed in routine follow-up clinical examination. In conclusion, the recurrence pattern may suggest that patient-initiated follow-up (PIFU) could be considered a potential alternative to clinical-based follow-up for HGEEC survivors, especially for patients without cervical involvement and after two years from treatment. Additional caution is needed in patients with cervical stromal involvement.

## 1. Introduction

Worldwide, 435,000 women are diagnosed and 91,600 die each year from endometrial cancer, the most common gynecological malignancy [1]. The incidence of endometrial cancer has been rising in high and middle income countries, secondary to increased obesity and aging of the population [2,3]. Historically, endometrial cancers were subdivided in two groups, Type I and Type II, according to their pathogenetic features [4] and are considered a surgically treatable disease [5]. Initially, endometrioid histology was considered Type I, but high grade endometrioid endometrial cancers (HGEEC) were gradually moved to the Type II group, as they shared immunohistochemical and prognostic features with non-endometrioid cancers [6]. The significant heterogeneity of HGEEC became more evident after the application of the molecular classification, as HGEEC were distributed among all subgroups of The Cancer Genome Atlas Program (TCGA) using the Proactive Molecular Risk Classifier for Endometrial Cancer (ProMisE) [7]. 

Previous studies provide conflicting data regarding HGEEC prognosis, with some showing similar [8] and others better [9,10] outcomes compared to non-endometrioid cancers and there are well-known disagreements regarding prognostic features of HGEEC [11,12]. Moreover, the diffusion of the ProMisE classification is not widespread and fully implemented worldwide; indeed, it requires adequate technology and logistics and hence cannot be used daily to support clinical choices in all the settings yet [13].

Considering these elements, our retrospective analysis aims to investigate independent risk factors for relapse and mortality in HGEEC and extrapolate recurrence patterns. 

## 2. Materials and Methods

We included all patients with high grade endometrioid endometrial adenocarcinoma treated across the Thames Valley Cancer Alliance Network (five recruiting sites) between March 2010 and January 2020. According to the literature, endometrioid adenocarcinomas with more than 50% solid architecture or 6–50% solid architecture and diffuse marked nuclear atypia were classified as HGEEC [14]. Patients with concomitant second primary cancer and patients that did not have primary surgical treatment were excluded from our cohort; similarly, we excluded those patients that received inadequate surgery due to a missed preoperative correct diagnosis [15].

All data were extracted retrospectively from electronic records of patients in the context of service evaluation for endometrial cancer. The service evaluation protocol was registered in accordance with the Oxford University Hospitals Trust requirements (registration number 5832) [16]. The design, analysis, interpretation of data, drafting and revisions conform to the Helsinki Declaration, the Committee on Publication Ethics guidelines (http://publicationethics.org/, accessed on 10 January 2023) and the Reporting of studies Conducted using Observational Routinely collected health Data (RECORD) Statement validated by the Enhancing the Quality and Transparency of Health Research Network (www.equator-network.org, accessed on 10 January 2023) [17]. The data collected were anonymized, considering the observational nature of the study, without personal data that could lead to formal identification of the patient. Each patient in this study was informed about the procedures and signed consent to allow data collection and analysis for research purposes. The study was not advertised. No remuneration was offered to the patients to give consent to be enrolled in this study.

Patients’ demographics and comorbidities were recorded. The Age-Adjusted Charlson Comorbidity Score (AACCS) was calculated and patients were divided in three groups: 0–1, 2–3 and >3 [18]. We extracted all the final histopathological features including FIGO Stage [19], depth of myometrial invasion (<50% and ≥50%), cervical stromal involvement, serosal breaching, adnexal, parametrial and pelvic lymph node involvement and the presence of distant metastases. The presence of lymphovascular invasion (LVSI) was also recorded. All tumors were classified according to the European Society of Gynaecological Oncology (ESGO)—European Society for Radiotherapy and Oncology (ESTRO)—European Society of Pathology (ESP) risk stratification model [20]. 

Treatment details regarding mode of surgery (laparotomy or laparoscopy), bilateral pelvic lymphadenectomy (BPLND) and administration of adjuvant treatment were collected. BPLND and adjuvant therapy were offered to all patients, and the reason why some did not proceed with the proposed treatment was also recorded. 

Surgical staging was performed according to national guidelines and in laparoscopic cases, we did not use uterine manipulator. All of the handling of the uterus was performed avoiding the cervix and a swab on a stick in the vagina or a vaginal tube was used during colpotomy. Follow-up was purely clinical-based under Gynaecological Oncologists and/or Clinical Oncologists in three months’ intervals for the first year, four months’ intervals for the second, biannually for the third and annually thereafter for two more years. 

We used independent samples *t*-test to compare continuous variables and Pearson chi-square or Fisher’s extract test for categorical variables. Survival rates were calculated from Kaplan–Meier curves and compared using log-rank tests. Univariate and multivariate Cox proportional hazards analysis was conducted to assess the potential risk factors for relapse and mortality. All statistical analyses were performed using IBM^©^SPSS Statistics 22.0. Statistical significance was considered for *p* < 0.05. 

## 3. Results

During the study period, 863 women underwent surgery for endometrial cancer in our center. HGEEC was confirmed as the only primary tumor on final histology in 96 patients, which represents 11.12% of all patients and 25.53% of all high-grade tumors.

The mean age of the patients was 68.96 years (range 47–93). A total of 82.3% were treated laparoscopically and 81.3% had pelvic lymph node dissection with 15 lymph nodes dissected on average (range 1–29). In 75% of HGEEC the disease was treated at an early stage (Stages I and II) and 25% had advanced disease (Stages III and IV) after surgical staging. Clinical, pathological and treatment details are summarized in Table 1.

**Figure 1:**
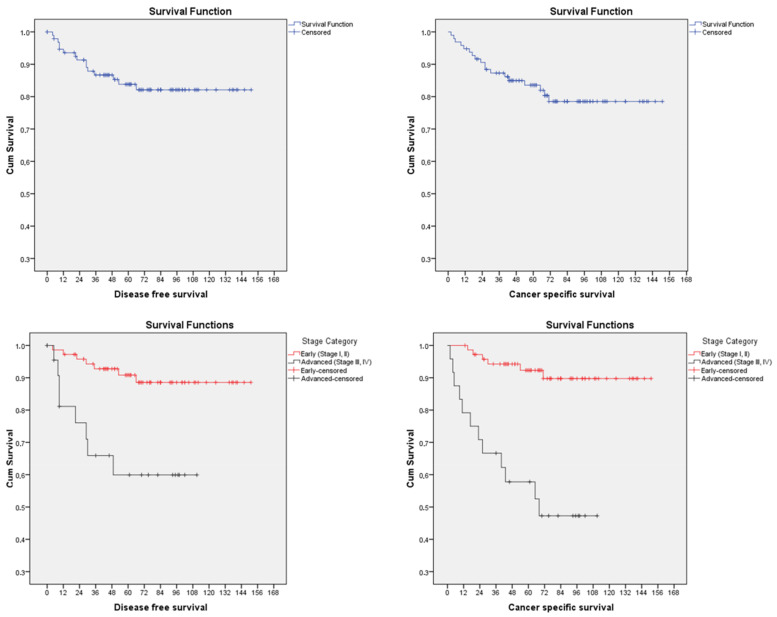
Disease-free (**left**) and cancer-specific survival (**right**) for patients with high grade endometrioid endometrial (cumulative with blue, early stage with red and advanced stage with black line).

The median follow-up after surgery was 68 months (range 2–151 months). Five-year overall and cancer-specific survival was 77.8% and 83.6%, respectively. Five-year disease-free survival was 83.8% (Figure 1). 

The recurrence rate in our cohort was 16%; more than half of the recurrences (53.33%) occurred within the first two years after surgery and 80% within three years. In only one of our cases (6.66% of recurrences), cancer relapsed more than 5 years from staging surgery (after 66 months). Mean surgery to recurrence and recurrence to death intervals were 25.4 (range 4–66) and 12.86 (range 1–35) months, respectively. 

Single vaginal vault recurrence was diagnosed only in one case (6.66% of recurrences), whereas in 60% of recurrence cases disease relapsed in the lungs; again, only one patient recurred in the para-aortic area with indeed negative pelvic lymph nodes. A total of 81.81% of the recurrences presented with symptoms and not a single recurrence was diagnosed in routine follow-up clinical examination of asymptomatic patients. The most common symptoms of recurrence were respiratory (3 cases) and gastrointestinal (3 cases). Two cases presented with loss of weight, two with bleeding and one with renal impairment. 

Among the seven early-stage (I and II) cases of our study group that had a recurrence, only one had isolated vaginal vault disease, three had only distant recurrence with no vaginal vault involvement and none of the seven recurrences were diagnosed in clinical examination before becoming symptomatic. The recurrence rate was 33.33% among stage III and IV cases and all the recurrences had a distant component. In 3/8 cases, there was concomitant vaginal vault disease, but even in these cases the diagnosis was made with imaging and not with clinical examination on regular follow-up (Table 2).

Univariate Cox proportional hazards analysis shows that the risk of recurrence is related to advanced stages (HR = 4.72; 95%CI 1.71–13.04; *p* = 0.003), cervical stromal involvement (HR = 7.11; 95%CI 2.56–19.76; *p* < 0.001) and pelvic lymph node involvement (HR = 4.58; 95%CI 1.33–15.78; *p* = 0.016). The risk of cancer-related death is lower in patients who underwent BPLND (HR = 0.35; 95%CI 0.13–0.94; *p* = 0.038) and higher with advanced stages (HR = 7.53; 95%CI 2.82–20.08; *p* < 0.001), deep myometrial invasion (HR = 3.17; 95%CI 1.13–8.9; *p* = 0.029), cervical stroma involvement (HR = 5.59; 95%CI 2.19–14.26; *p* < 0.001), serosal breaching (HR = 7.77; 95%CI 3.00–20.12; *p* < 0.001), parametrial involvement (HR = 5.08; 95%CI 1.80–14.30; *p* = 0.002), pelvic lymph node involvement (HR = 5.27; 95%CI 1.66–16.70; *p* = 0.005), distant metastases (HR = 5.93; 95%CI 1.36–25.90; *p* = 0.018) and high risk features according to ESGO–ESTRO–ESP stratification (HR = 5.05; 95%CI 1.42–17.91; *p* = 0.012) (Table 3).

**Figure 2:**
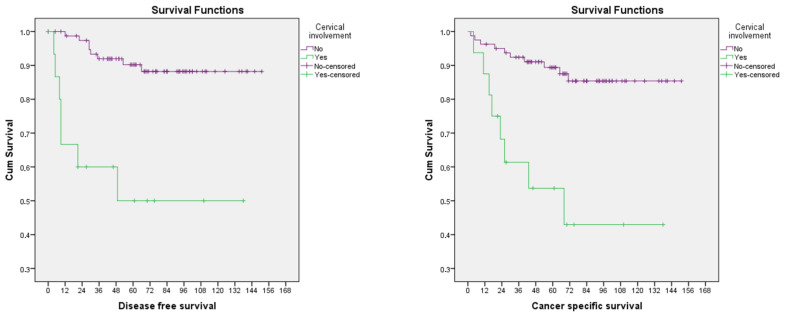
Disease-free (**left**) and cancer-specific survival (**right**) for patients with high grade endometrioid endometrial cancer with (green line) and without (purple line) cervical stromal involvement.

However, at multivariable Cox analysis, only cervical involvement is confirmed as independently related with a higher risk of recurrence (HR = 25.67; 95%CI 2.95–223.30; *p* = 0.003) and cancer-related death (HR = 15.39; 95%CI 1.29–183.43; *p* = 0.031) after adjusting for age, comorbidities (AACCS), surgical approach, BPLND depth of myometrial invasion, adnexal involvement, serosal breaching, parametrial involvement, pelvic lymph node involvement, distant metastases, LVSI and administration of adjuvant treatment. Cervical stroma involvement has a significant impact to 5-year disease-free survival (50% vs. 90.2%, *p* < 0.001) and 5-year cancer-specific survival (53.7% vs. 89.4%, *p* < 0.001) (Figure 2.). 

Although it is difficult to compare the recurrence pattern of patients with and without cervical involvement due to the very small sample, it is obvious that patients with cervical involvement tend to relapse earlier compared to those with clear cervix (mean and median treatment to recurrence time is 15 and 9 months vs. 34.5 and 29.5, respectively). Moreover, all the patients with cervical involvement who had a recurrence had distant and/or multifocal disease at presentation. 

## 4. Discussion

In our cohort, the recurrence rate was 16%, which is lower compared to the majority of previous reports (19.6–23.6%) [11,21,22]. Wang et al. [12] reported a 13.7% recurrent rate, but in that study, early-stage (I and II) tumors accounted for 82.1% of the patients, which is higher compared to the 75% observed in our series. In terms of survival, our outcomes were comparable to the literature; 77.8% vs. 70.8% [12] and 76.8% [23] 5-year overall survival, and 83.6% vs. 81.9% [12] 5-year cancer-specific survival. Our favorable outcomes could be explained by the efficient surgical management (BPLND in 81.3% of cases with 15 lymph nodes removed on average) and the high rate of adjuvant treatment (83.1% of cases). 

Although univariate Cox proportional hazards analysis demonstrated that outcomes of HGEEC were related to almost all of the known clinicopathological risk factors described in the literature for endometrial cancer [24], only cervical involvement is independently related to recurrence and cancer-specific survival. However, prognostic risk factors for endometrial cancer have been extrapolated from cohorts with heterogeneous histological types, and very few studies have focused on only high grade endometrioid histology. 

Wang et al. reviewed a group of 117 patients with HGEEC and showed that myometrial invasion ≥ 50% was an independent prognostic factor for both survival and recurrence [12]. Conversely, Rasool et al. found no relationship between depth of myometrial invasion and clinical outcomes in their cohort of 176 early-stage HGEEC patients [25], and Zhu et al. confirmed these findings in their cohort that included high grade endometrioid cases of all stages [23]. Despite the controversial evidence for HGEEC, depth of myometrial invasion has been considered an independent risk factor for metastatic disease, relapse and cancer-specific mortality in endometrial cancer in general [24,26]. In our cohort, depth of myometrial invasion ≥50% carries a more than threefold higher risk of cancer-related mortality (*p* = 0.029), although that relationship disappears after adjusting for age, AACCS, surgical approach, pelvic lymph node assessment, depth of myometrial invasion, adnexal involvement, serosal bridge, parametrial involvement, pelvic lymph node involvement, distant metastases, LVSI and administration of adjuvant treatment. 

According to Wang et al., adnexal involvement is another independent prognostic factor in HGEEC [12]. On the one hand, overall survival has also been proved to be related with adnexal involvement by the tumor in a small cohort of 85 HGEEC patients [23]. On the other hand, our data suggest that there is no significant relationship between adnexal metastases and recurrence (*p* = 0.235) or cancer-specific mortality (*p* = 0.078). 

Interestingly, cervical stromal involvement was the only independent pathological risk factor in our study, affecting both 5-years disease-free (50% vs. 90.2%, *p* < 0.001) and cancer-specific survival (53.7% vs. 89.4%, *p* < 0.001). Wang et al. showed a 3.25 fold increased risk of recurrence but no difference in mortality for patient with HGEEC extending to the cervical stroma [12]. Zhu et al. also failed to prove a significant relationship between cervical involvement and survival in HGEEC [23]. These discrepancies might be attributed to the small sample size of the studies. 

The significance of the cervical involvement in prognosis of HGEEC further highlights the paramount importance of a correct evaluation of cervical invasion in patients with endometrial cancer, especially by 3D transvaginal sonography which demonstrated good diagnostic accuracy in terms of sensitivity and specificity compared with magnetic resonance imaging [27]. It also depicts the importance of minimal cervical manipulation during surgical staging. Although minimally invasive approaches are considered safe in early-stage endometrial cancer even if the cervix is involved [16], the use of uterine manipulators should be avoided in all cases as it seems to be related with adverse outcomes [28]. 

The timing of recurrence in our cohort is in agreement with a systematic review that showed that 68–100% of recurrences occur within three years from primary treatment [29]. These findings justify the rationale of more frequent follow-up for the first 2–3 years that has been traditionally proposed [30,31].

The traditional practice of regular clinical follow-up appointments for speculum and bimanual examination is not evidence-based and is based on the assumption that early detection of disease is important if the disease is confined to the vaginal vault [31], which is considered to be the most common site of recurrence of early-stage endometrial cancer [32]. However, as described in our results, this is not the case for HGEEC. Our findings are consistent with previous studies which show that in early-stage HGEEC, the recurrences are rarely confined to the vault, namely 1/8 was confined to the vault according to Kato et al. [11] and 5/26 according to Gayar et al. [22]. Similarly, Kato et al. found no evidence of vaginal disease among the cases that developed a recurrence after treatment for stage III HGEEC [11], which is in agreement with our findings.

Our results confirm the literature that suggests that in HGEEC, single-vault recurrence is rare (5–10% of recurrences) [11,12,21]. We have also observed that in the vast majority of the cases (81.81%), the recurrence gives symptoms that trigger imaging. Even in the asymptomatic cases, the diagnosis of recurrence was made on CT scan and not clinically. Hence, the traditional clinical-based close follow-up [31] appears ineffective in the case of HGEEC. Moreover, concerns have been raised that routine follow-up does not meet the psychological needs of survivors [33] and might delay diagnosis and treatment in case of symptomatic relapse [34]. Finally, routine clinical follow-up for endometrial cancer puts a substantial pressure to healthcare systems (23,619 follow-up appointments for endometrial cancer in 2020–2021 in the UK [35]), with no clear evidence of outcomes’ improvement [29]. 

Based on the above data, patient-initiated follow-up (PIFU) seems a reasonable option for patients treated for HGEEC without cervical involvement. The British Gynaecological Cancer Society has already incorporated PIFU in the follow-up scheme for HGEEC but only 2 years after the end of treatment [30]. However, only 58% of cancer centers and units in the UK offer PIFU for HGEEC at stage IA and less than 50% for more advanced disease [36]. For patients with cervical involvement who are at increased risk of early multifocal recurrence, regular imaging can be considered; however, the cost-effectiveness of such an approach is yet to be proven. 

The small number of our cohort and the retrospective design are the main limitations of our study. The hazard ratios need to be interpreted with caution because of the wide confidence intervals secondary to the small sample size. Moreover, no data regarding ProMisE classification and any further molecular assessment were systematically available for all the patients [37,38]. However, this is one of the very few studies that have focused only on HGEEC. We provide detailed information on treatment and a very comprehensive description of recurrence patterns. In the future, it would be of utmost importance to incorporate molecular assessment and to integrate machine learning models to predict the prognosis [39,40]. 

## 5. Conclusions

In conclusion, our study suggests that cervical stromal involvement is the only risk factor for recurrence and cancer-specific mortality for HGEEC after adjusting for other pathological and treatment parameters. The recurrence pattern may suggest that PIFU could be considered a potential alternative to clinical-based follow-up, especially for patients without cervical involvement after two years from treatment. 

## Figures and Tables

**Table 1:** Clinicopathological characteristics and treatment details of patients with HGEEC.

	N (%)	Recurrences		Cancer-Related Deaths	
		N	*p*-Value	N	*p*-Value
**AGE**			0.377		0.139
<65	30 (31.3)	3		3	
≥65	66 (68.8)	12		15	
**AACCS**			0.593		0.552
0–1	9 (9.4)	1		1	
2–3	59 (61.5)	8		10	
>3	28 (29.2)	6		7	
**Surgical approach**			0.726		0.301
Laparoscopy	79 (82.3)	12		13	
Laparotomy	17 (17.7)	3		5	
**Pelvic lymph node dissection**			0.471		0.097
No	18 (18.8)	4		6	
Yes	78 (81.3)	11		12	
**Adjuvant treatment**			0.449		0.729
No	15 (16.9)	1		3	
Yes	74 (83.1)	13		13	
**FIGO Stage**			0.179		0.02 *
IA	41 (42.7)	3		3	
ΙΒ	26 (27.1)	3		2	
ΙΙ	5 (5.2)	1		1	
ΙΙΙA	5 (5.2)	2		2	
ΙΙΙΒ	6 (6.3)	1		3	
ΙΙΙC1	7 (7.3)	3		4	
IIIC2	3 (3.1)	1		1	
IVA	0 (0)	0		0	
IVB	3 (3.1)	1		2	
**Depth of myometrial invasion**			0.135		0.028 *
<50%	49 (51)	5		5	
≥50%	47 (49)	10		13	
**Cervical stroma involvement**			0.003 *		0.002 *
No	80 (83.3)	8		10	
Yes	16 (16.7)	7		9	
**Adnexal involvement**			0.235		0.078
No	90 (93.8)	13		15	
Yes	6 (6.3)	2		3	
**Serosal breach**			0.370		0.010 *
No	85 (88.5)	12		11	
Yes	11 (11.5)	3		7	
**Parametrial involvement**			0.653		0.019 *
No	86 (89.6)	13		13	
Yes	10 (10.4)	2		5	
**Pelvic lymph node involvement**			0.043 *		0.010 *
No	67 (85.9)	7		7	
Yes	11 (14.1)	4		5	
**Distant metastases**			0.403		0.089
No	93 (96.9)	14		16	
Yes	3 (3.1)	1		2	
**LVSI**			0.736		0.716
No	41 (42.7)	7		7	
Yes	55 (57.3)	8		11	
**ESGO–ESTRO–ESP Risk stratification**			0.02 *		<0.001 *
Intermediate	24 (25)	3		3	
High-intermediate	48 (50)	4		3	
High	24 (25)	8		12	

* for statistically significant results.

**Table 2:** Cases of recurrent high grade endometrioid endometrial and their characteristics.

Stage	Myometrial Invasion	Cervical Involvement	Adnexal Involvement	Serosal Breach	Parametrial Involvement	Pelvic LN Involvement	Paraaortic LN Involvement	Distant Metastasis	LVSI	EBRT	VBT	CT	Presenting Symptom at Recurrence	Site of Recurrence	Treatment for Recurrence	DFS (Months)	Survival after Recurrence (Months)
IA	<50	No	No	No	No	No		No	No	No	Yes	No	Weight loss and fatigue	Lung, kidney and brain	BSC	66	5
IA	<50	No	No	No	No	No		No	No	No	Yes	No		Vault, sigmoid colon, pelvic lymph nodes and lung	HT + EBRT	35	19
IA	<50	No	No	No	No	No		No	No	No	Yes	No	Vaginal bleeding	Vault, pelvic bones, lung	BSC	22	4
IB	≥50	No	No	No	No	No		No	Yes	Yes	Yes	No	Rectal bleeding	Lung	CT	53	
IB	≥50	No	No	Νο	No			No	No	No	No	No	Hypoxia, vomiting and loss of weight	Vault	BSC	29	1
IB	≥50	No	No	No	No			No	No	Yes	No	No		Lung	EBRT	12	7
II	≥50	Yes	No	No	No	No		No	Yes	Yes	No	No	Shortness of breath	Vault, peritoneum, lung, liver	EBRT + CT	4	11
IIIA	≥50	No	No	Yes	No			No	Yes			Yes	Asymptomatic (Unable to examine, hence CT)	Vault, pelvic lymph nodes, anterior abdominal wall	CT	29	11
IIIA	<50	Yes	Yes	No	No	No		No	Yes	No	Yes	Yes		Vault, liver, bones	HT	21	2
IIIB	≥50	Yes	No	Yes	Yes	No		No	No	Yes	No	No		Multifocal peritoneal deposits	BSC	8	3
IIIC1	≥50	Yes	No	No	No	Yes		No	No	Yes	Yes	Yes	Right upper abdominal pain	Multifocal upper abdominal intra-abdominal nodules	HT + EBRT + CT	49	19
IIIC1	≥50	No	No	No	No	Yes		No	Yes				Right iliac fossa pain	Lung	CT	30	35
IIIC1	<50	Yes	No	No	No	Yes		No	Yes	Yes	Yes	No	Hypoxia	Lung	CT	9	8
IIIC2	≥50	Yes	No	No	No		Yes	No	Yes	No	No	Yes	Asymptomatic (FU scan after radiotherapy)	1st: Para-aortic lymph nodes, 2nd: Lung (11 months after 1st recurrence)	EBRT (for 1st recurrence) HT + CT (for 2nd recurrence)	9	34
IVB	≥50	Yes	Yes	No	Yes	Yes		Yes	Yes	No	No	Yes	Acute kidney injury	Vault, sigmoid colon, pelvic lymph nodes	CT	5	21

LN = lymph nodes, EBRT = external beam radiotherapy, VBT = vault brachytherapy, CT = chemotherapy, HT = hormonotherapy, BSC = best supportive care, DFS = disease free survival.

**Table 3:** Univariate Cox proportional hazards analysis for the risk of recurrence and disease-specific death for high grade endometrioid endometrial cancer.

	Recurrence		Cancer-Specific Death	
	HR (95% CI)	*p*-Value	HR (95% CI)	*p*-Value
**AGE**	1.04 (0.98–1.11)	0.192	1.05 (0.99–1.11)	0.066
**MDT to Theatre interval**	1.01 (0.99–1.02)	0.653	1.01 (0.99–1.02)	0.471
**AACCS**				
0–1				
2–3	1.24 (0.16–9.89)	0.842	1.53 (0.20–12.01)	0.684
>3	2.03 (0.24–16.81)	0.513	2.36 (0.29–19.21)	0.422
**Surgical approach**				
Laparoscopy				
Laparotomy	1.42 (0.40–5.02)	0.591	2.18 (0.78–6.12)	0.140
**Pelvic lymph node dissection**				
No				
Yes	0.46 (0.15–1.46)	0.190	0.35 (0.13–0.94)	0.038 *
**Number of LN removed**	0.95 (0.87–1.03)	0.215	0.95 (0.88–1.03)	0.201
**Adjuvant treatment**				
No				
Yes	2.25 (0.30–17.25)	0.434	0.72 (0.21–2.53)	0.608
**FIGO Stage**				
IA				
ΙΒ	1.67 (0.34–8.28)	0.530	1.12 (0.19–6.68)	0.904
ΙΙ	4.25 (0.44–41.06)	0.211	3.77 (0.39–36.41)	0.251
ΙΙΙA	6.17 (1.03–36.99)	0.046 *	5.82 (0.97–34.84)	0.054
ΙΙΙΒ	3.75 (0.39–36.12)	0.253	10.59 (2.13–52.69)	0.004 *
ΙΙΙC1	8.03 (1.61–40.06)	0.011 *	10.19 (2.27–45.72)	0.002 *
IIIC2	6.19 (0.64–59.70)	0.115	5.25 (0.55–50.62)	0.151
IVB	11.09 (1.15–107.02)	0.038 *	15.20 (2.53–91.30)	0.003 *
**Stage category**				
Early (I-II)				
Advanced (III-IV)	4.72 (1.71–13.04)	0.003 *	7.53 (2.82–20.08)	<0.001 *
**Depth of myometrial invasion**				
<50%				
≥50%	2.53 (0.86–7.40)	0.091	3.17 (1.13–8.90)	0.029 *
**Cervical stroma involvement**				
No				
Yes	7.11 (2.56–19.76)	<0.001 *	5.59 (2.19–14.26)	<0.001 *
**Adnexal involvement**				
No				
Yes	2.94 (0.66–13.06)	0.155	3.37 (0.98–11.65)	0.055
**Serosal breach**				
No				
Yes	3.11 (0.87–11.04)	0.080	7.77 (3.00–20.12)	<0.001 *
**Parametrial involvement**				
No				
Yes	2.21 (0.50–9.79)	0.298	5.08 (1.80–14.30)	0.002 *
**Pelvic lymph node involvement**				
No				
Yes	4.58 (1.33–15.78)	0.016 *	5.27 (1.66–16.70)	0.005 *
**Distant metastases**				
No				
Yes	4.43 (0.58–33.70)	0.150	5.93 (1.36–25.90)	0.018 *
**LVSI**				
No				
Yes	0.93 (0.34–2.57)	0.890	1.25 (0.49–3.23)	0.642
**ESGO–ESTRO–ESP Risk stratification**				
Intermediate				
High–intermediate	0.68 (0.15–3.04)	0.614	0.51 (0.10–2.50)	0.402
High	3.72 (0.99–14.05)	0.053	5.05 (1.42–17.91)	0.012 *

* for statistically significant results, HR = hazard ratio, MDT = multidisciplinary team discussion.

## Data Availability

Data is unavailable due to privacy restrictions.
